# Diagnostic Potential of the NMDA Receptor Peptide Assay for Acute Ischemic Stroke

**DOI:** 10.1371/journal.pone.0042362

**Published:** 2012-07-27

**Authors:** Svetlana A. Dambinova, Kerstin Bettermann, Theodore Glynn, Matthew Tews, David Olson, Joseph D. Weissman, Richard L. Sowell

**Affiliations:** 1 WellStar College of Health and Human Services, Kennesaw State University, Kennesaw, Georgia, United States of America; 2 Department of Neurology, Penn State Milton S. Hershey Medical Center, Penn State College of Medicine, Hershey, Pennsylvania, United States of America; 3 Department of Emergency Medicine, Michigan State University Emergency Medicine, Residency, Ingham Regional Medical Center, Lansing, Michigan, United States of America; 4 DeKalb Medical, Decatur, Georgia, United States of America; Georgia Health Sciences University, United States of America

## Abstract

**Background:**

The acute assessment of patients with suspected ischemic stroke remains challenging. The use of brain biomarker assays may improve the early diagnosis of ischemic stroke. The main goal of the study was to evaluate whether the NR2 peptide, a product of the proteolytic degradation of *N*-methyl-D-aspartate (NMDA) receptors, can differentiate acute ischemic stroke (IS) from stroke mimics and persons with vascular risk factors/healthy controls. A possible correlation between biomarker values and lesion sizes was investigated as the secondary objective.

**Methods and Findings:**

A total of 192 patients with suspected stroke who presented within 72 h of symptom onset were prospectively enrolled. The final diagnosis was determined based on clinical observations and radiological findings. Additionally gender- and age-matched healthy controls (n = 52) and persons with controlled vascular risk factors (n = 48) were recruited to compare NR2 peptide levels. Blinded plasma was assayed by rapid magnetic particles (MP) ELISA for NR2 peptide within 30 min and results for different groups compared using univariate and multivariate statistical analyses. There was a clinical diagnosis of IS in 101 of 192 (53%) and non-stroke in 91 (47%) subjects. The non-stroke group included presented with acute stroke symptoms who had no stroke (n = 71) and stroke mimics (n = 20). The highest NR2 peptide elevations where found in patients with IS that peaked at 12 h following symptom onset. When the biomarker cut off was set at 1.0 ug/L, this resulted in a sensitivity of 92% and a specificity of 96% to detect IS. A moderate correlation (r_s_ = 0.73) between NR2 peptide values and acute ischemic cortical lesions (<200 mL) was found.

**Conclusions:**

This study suggests that the NR2 peptide may be a brain specific biomarker to diagnose acute IS and may allow the differentiation of IS from stroke mimics and controls. Additional larger scale clinical validation studies are required.

## Introduction

About 20–30% of all patients admitted to the emergency department (ED) are having stroke-like symptoms as reported in hospital based studies [1], [2], while more selective criteria [3] showed that above the age of 50 years, stroke mimics are very rare (3%). The diagnosis of IS is largely based on clinical assessment and neuroimaging. However, up to 40% of all acute stroke patients cannot have an emergent brain MRI [4], [5], either because it is contraindicated or unavailable. Timely and accurate identification of IS patients is essential for optimal patient triaging and choice of treatment strategy [6]. A rapid and affordable assay that can detect brain-borne biomarkers in blood following IS, and that allows to differentiate IS from non-stroke could potentially reduce the cost of unnecessary admissions and diagnostic workups.

Currently, a number of biomarkers for stroke diagnosis are being investigated in clinical studies, including in the emergency room (ER) and in- and outpatient settings [7]. However, no biomarker(s) has yet proven to be effective at differentiating IS from non-stroke. In addition, blood assays detecting brain biomarkers may enhance the ability to detect acute stroke in the presence of prior strokes [8].

This study examined the diagnostic potential of the NR2 peptide assay to differentiate acute IS from stroke mimics and controls when used in conjunction with neurological assessments and MRI/CT imaging. The secondary objective was to explore a possible correlation between biomarker values and stroke lesion volumes in stroke patients presenting within 72 h of symptom onset.

## Methods

This was a prospective, blinded study performed from January 2005 to March 2011 at two clinical sites. The local Investigative Research Board Committees approved the study, and written informed consent was obtained from each enrolled subject or a family member. Patients with suspected TIA or acute stroke were admitted to the Emergency Department (ED) at DeKalb Medical Center (Decatur, GA) and at Ingham Regional Medical Center, Michigan State University (Lansing, MI). Additionally individuals with vascular risk factors and healthy controls were recruited at Penn State College of Medicine (PSU) and Kennesaw State University (KSU). All controls besides informed consent completed a standard questionnaire detailing medical history, stroke risk factors, medication use, and history of any prior strokes.

### Subjects

Adults, 18 years or older with suspected TIA (defined as a neurological deficit that resolved within 24 h), or acute stroke presenting within 72 h of symptom onset were included in the study. Patients were excluded if they were pregnant, or had been transferred from an inpatient care facility.

**Figure 1:**
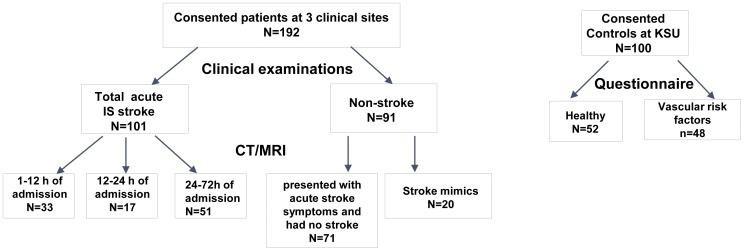
Flow diagram of study population: (i) patients with definite acute ischemic stroke (IS) and TIA, (ii) the non-stroke group included patients presented with acute stroke symptoms and had no stroke and stroke mimics, (iii) control group comprising healthy volunteers and persons with controlled vascular risk factors (hypertension, diabetes mellitus, and heart disease).

The study included a total of 460 subjects recruited as a convenience population: there were n = 360 patients enrolled in to the stroke study, 52 healthy controls, and 48 individuals with vascular stroke factors. In the stroke study population 93 patients were excluded as they did not undergo emergent CT/MRI imaging due to metal implants, medical instability, obesity or severe claustrophobia. Patients with intracerebral hemorrhage (n = 10) and IS patients without known time of symptom onset or time last known being well (n = 36) were excluded as well. Severely hemolyzed plasma samples withdrawn from 29 TIA/stroke patients could not be analyzed reliably and those subjects were excluded from the study. Data from 292(63%) study participants have been included in the final analysis of the study (Fig. 1).

### Clinical and radiological procedures

All patients who presented with acute stroke symptoms within the study time window underwent a standard neurological and general medical evaluation and assessment using the National Institutes of Health Stroke Scale (NIHSS). Past medical history and medication history were obtained and a head CT or brain MRI was performed within 24 h. Each patient was seen and followed during hospitalization by an experienced stroke neurologist. Hospital course and discharge examination data were also noted. History of prior strokes was based upon history, documented in the medical records, and findings on imaging studies.

Standard MRI included axial fluid attenuation recovery (FLAIR), T1- sagittal, T2-weighted axial, diffusion weighted imaging (DWI), and apparent diffusion coefficient maps that a board certified neuroradiologist at each site read and interpreted. Stroke location and ischemic stroke volumes were reported [9], and then reports were uploaded to each subjects' file. MRI images were obtained on a clinical MRI scanner (Siemens 1.5 Tesla Avanto) operating with single-shot echo planar-capable gradients. General Electronics (GE, USA) Centricity software was used for image analysis.

Infarct volumes were measured using computer-assisted volumetric software (Analyze software 6.1; Biomedical Imaging Resource, Rochester, Minn). A pen-trace method was used to outline the region of interest (i.e., area of acute stroke) on each axial image and the total volume was reported in mL. In cases of multifocal infarction, the sum of the individual volumes was used.

### Sample collection and storage

This study involved a single blood draw from each recruited participant after clinical determinations were performed. Blood samples (5 mL) were drawn by venipuncture into vacuum tubes containing sodium EDTA (Becton Dickinson) placed on ice. Plasma drawn from each participant was separated by centrifugation at 3,000 rpm for 5 min at 4°C. Sample processing was completed within 30 min of blood collection to protect the NR2 peptide from depletion by endogenous serine proteases. Hemolyzed plasma was not acceptable due to cross-reaction with hemoglobin.

Processed samples were stored as multiple 0.5-mL aliquots (n = 6) at −80°C before delivery to KSU Lab.

### NR2 peptide detection in plasma

Aliquot from each batch of samples (n = 30) was analyzed for NR2 peptide according to the manufacturer's manual (Gold Dot Test, CIS Biotech, Inc., Atlanta, GA) by an investigator blinded to the clinical and imaging data. Once aliquot was thawed for testing and used, remaining amounts were discarded.

Briefly, 20 uL plasma samples, five calibrators, negative/positive controls in duplicates, and 80 uL of working mixture consisting of magnetic particles (MP) with covalently attached specific antibodies against NR2 peptide were added to the microtiter plate. The mixture was incubated for 2 min at 37°C; NR2 antibodies labeled horseradish peroxidase (HRP) solution was then added for 20 min at 37°C. After the bound magnetic particles were washed with a buffer using a magnetic separator, the reaction was revealed by pipetting 100 uL ready-to-use TMB substrate into each well of a microtiter plate. The color reaction was developed for 8 min at 25°C, stopped with acid solution (100 uL), and monitored at 490/630 nm on a microplate reader (Bio Tek ELx800™, Fisher Sci.).

The NR2 peptide concentrations in plasma were determined by plotting their absorbance values on a calibration curve constructed from the absorbance units of each calibrator and their known concentrations. Assay results were filed by the Lab director into a restricted access database. The intra-assay coefficient variation (CV) was 5.3%–6.3%, and the interassay CV was 5.9%–9.8%.

### Statistical analysis

To distinguish between the stroke and non-stroke, definite and probable strokes (TIA) were classified as stroke, whereas definite non-stroke and possible strokes (TIA) who had no evidence of stroke or TIA during the further diagnostic workup were referred as non-stroke.

Differences between groups were assessed using descriptive statistics and standard tests of significance. Univariate statistical analyses with 95% CIs were calculated. Analyses were performed on the number of subjects included in the study using R Statistical package (http://www.r-project.org/). Continuous independent variables were compared by use of the one-way ANOVA followed by post hoc test. The Spearman rank correlation test was used to analyze the association between NR2 peptide values and infarct volume measured on CT or MRI.

A receiver operator characteristic (ROC) curve was used to calculate the cutoff value for optimal sensitivity and specificity [10]. ROC curves (sensitivity vs. 1-specificity) for IS vs 3 different groups (stroke mimics presenting with acute stroke like symptoms and all controls) by varying the cut off value for distinguishing IS from non-stroke were built. The gold standard used for constructing the ROC curve was based on final diagnoses. We used the partial area under the ROC curve for the region with specificity between 0.75 and 0.95, as a global measure of the diagnostic effectiveness of the NR2 peptide assay [11]. To test the global null hypothesis that the NR2 peptide does not have adequate accuracy to differentiate IS from non IS, we evaluated if the average AUC is at least 0.8.

## Results

### Patients and control group characteristics

We studied 192 patients of 360 subjects included in the study with suspected acute IS prospectively enrolled at participating clinical sites within 72 h of symptom onset. Clinical assessments, diagnostic workup, and imaging confirmed the final diagnosis as definite stroke in 87(45%), definite TIA in 6(3%), and probable stroke in 8(4%). A definite non-stroke group comprised 20(10%) patients with stroke mimics (migraine, seizures, and meningitis) and 71 (37%) patients who presented with acute stroke symptoms but had no stroke (history of stroke and TIA >1 year prior to the study, n = 60, aneurism, n = 8, and palsies, n = 3): 45 (23%) as a possible stroke, 26 (14%) as a possible TIA (Table 1). The dichotomized final diagnosis was IS in 101 of 192(53%) and neurological deficits of other etiology in 91(47%).

**Table 1:** Characteristics of the study population.

Feature	IS n = 101	Non-Stroke n = 91	Controls	
			Healthy n = 52	Controlled vascular risk factors, n = 48
**Median age (range in years)**	62.0(26–95)	61.0(24–95)	59.0(29–92)	60(28–80)
**Male gender (%)**	54(53.5)	52(52.5)	28(53.8)	26(54.1)
**Median time of symptom onset**	8.2	9.0	-	-
**Number presenting**				
0–12 h	33	30	-	-
12–24 h	17	20	-	-
24–72 h	51	41	-	-
**Vascular risk factors/n (%)**				
Diabetes mellitus	23/101(23)	24/91(26)	-	4/48(8)
Hypertension	49/101(49)	40/91(44)	-	27/48(56)
Heart disease	21/101(21)	19/91(21)	-	0
**Past history of stroke/n (%)**	62/101(61)	57/91(63)	-	-
**Risk factors for stroke mimic/n (%)**				
Migraine	-	2/91(2)	-	-
Seizures	1/101(1)	3/91(3)	-	-
Meningitis	-	15/91(16)	-	-
**NIHSS**				
Mean	7.0	4.0	-	-
median	5.3	3.0	-	-
**Number with CT/MRI(%)**	101(100)	94(95)	-	-

Median NIHSS for patients with IS on admission was 5.3 (range, 1–20) with infarct volumes ranging from 1–250 mL. In the acute IS group, 43% and 57% of patients had cortical and subcortical strokes, respectively. Two patients admitted to the ED with TIA progressed to acute IS within 24 h of admission.

The control group included gender- and age-matched healthy individuals (n = 52) and persons with vascular risk factors (n = 48) without history of stroke. Characteristics of the study population subdivided into stroke, non-stroke, and controls are recorded in Table 1.

### NR2 peptide concentrations

**Figure 2:**
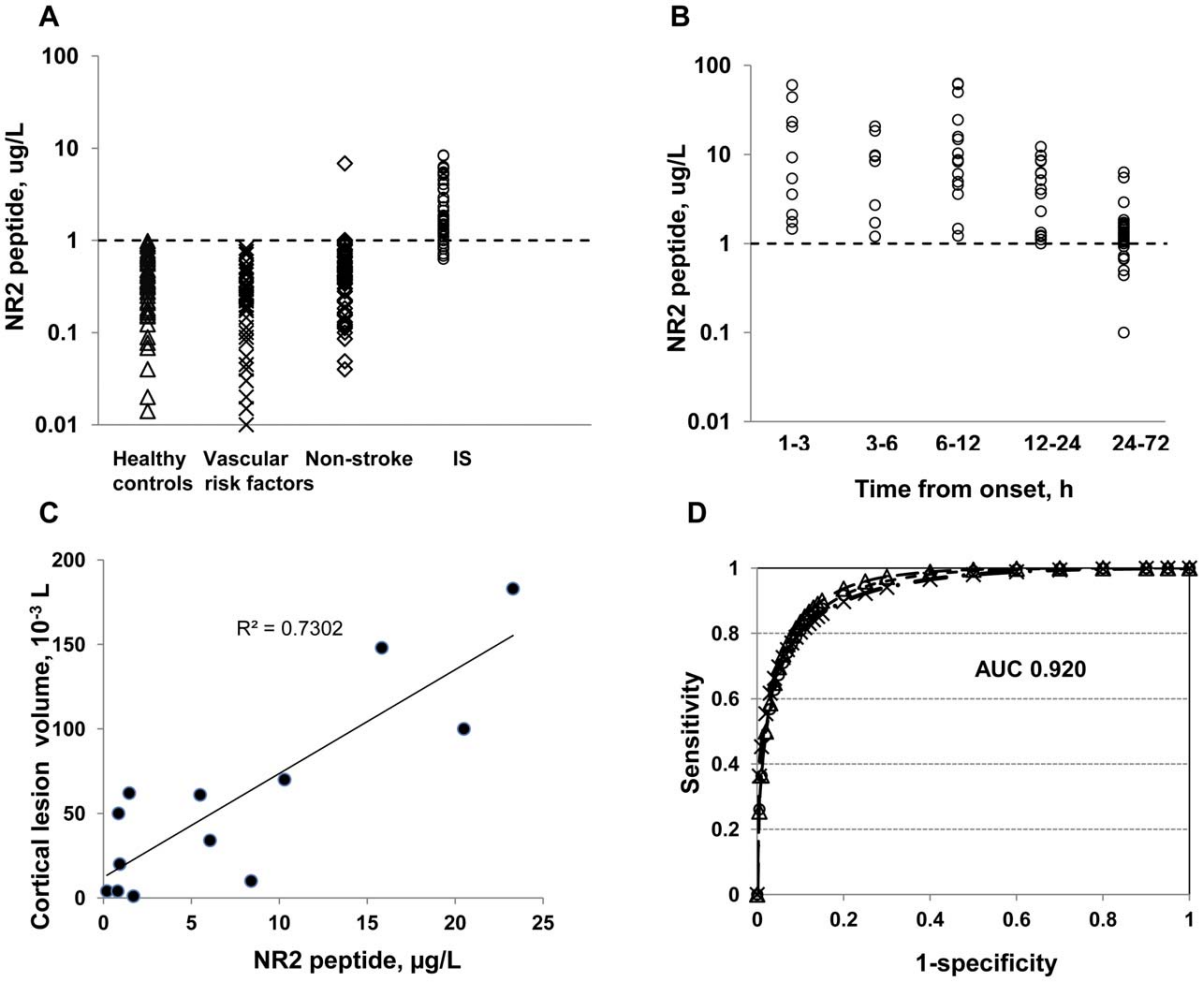
Distribution of plasma NR2 peptide concentrations in (A) healthy controls (n = 52), persons with controlled vascular risk factors (n = 48), non-stroke (n = 99) and acute ischemic stroke (n = 101). Distribution of NR2 peptide in plasma of patients with acute IS depending on time of symptom onset: n = 10 at 1–3 h, n = 8 at 3–6 h, n = 15 at 6–12 h, n = 17 at 12–24 h, and n = 51 at 24–72 h (**B**). Correlation of NR2 peptide concentrations with new cortical lesion (**C**). ROC curves for plasma NR2 peptide depended comparison group (**D**). Areas under the each curve are 0.930 calculated for the biomarker potential to distinguish acute IS vs certain control group, 0.914 for acute IS vs non-stroke, and 0.920 for acute IS vs combined control and non-stroke groups.

The NR2 peptide plasma concentrations for each group are shown in Fig. 2. There were no adverse events from performing the NR2 peptide assay. Medication use did not modify biomarker concentrations.

Ninety three patients with acute cortical and subcortical IS (definite stroke, n = 81, definite TIA, n = 6, and probable stroke, n = 6) had an increased NR2 peptide level with a median concentration of 5.44 ug/L (range, 0.1–62.71 ug/L). There were patients (n = 8) with acute IS (definite stroke n = 6 and probable stroke n = 2) and low NR2 peptide-concentrations (0.1–0.93 ug/L) with four of them having a recurrent stroke within 1–6 months prior to study enrollment. Healthy individuals had a median value of 0.33 ug/L (range, 0.02–1.15 ug/L), whereas a median concentration of 0.27 ug/L (range, 0.01–1.54 ug/L) was calculated for individuals with controlled vascular risk factors. Healthy individuals (n = 2) and persons with controlled vascular risk factors (n = 2) showed increased NR2 peptide values (1.10–1.54 ug/L).

Group comparison of mean values of the NR2 peptide concentration demonstrated significant differences (P<0.0001) for patients with acute IS compared to all control groups.

Analysis of the NR2 peptide distribution in patients with acute IS depending on the time of symptoms onset is shown in Fig. 2b. We observed the increased NR2 peptide values (1.46–5.38 ug/L) in 3 patients with subcortical lesions (about 1 mL size) within 1 h after the onset. The highest peptide concentrations (median 8.68 ug/L, range 1.20–62.71 ug/L) occurred within 1–12 h following symptom onset. There was a moderate to strong correlation (r_s_ = 0.73) between the size of acute ischemic lesions found on brain CT/MRI and NR2 peptide concentrations in acute IS patient for cortical lesions measuring <200 mL (Fig. 2c). There was no correlation found for the biomarker values and cortical lesion volumes beyond 200 mL (r_s_ = 0.21).

Operating characteristics of the NR2 peptide are depicted in Table 2. The predictive values and likelihood ratios at specific cutoff points were chosen to approximate a sensitivity of 92 and 98% and specificities of 72%, and 96%, respectively. The optimal cutoff value for acute IS diagnosis was 1.0 ug/L with a sensitivity of 92% and a specificity of 96% at which a positive predictive value of 93% for the NR2 peptide assay was achieved. Considering a prior probability of stroke (odds) of 34%, positive and negative post-test probabilities resulted in 93% (95% CI, 87% to 96%) and 4% (95% CI, 2%–8%) respectively.

**Table 2:** Operating characteristics of different cutoff points for NR2 peptide concentrations in all study groups.

Parameter	NR2 peptide test performance at respective cutoff values	
	0.5 ug/L	1.0 ug/L
Sensitivity, % (95% CI)	98.0 (93.0 to 99.8)	92.1 (85.0 to 96.5)
Specificity, % (95% CI)	71.9 (65.1 to 78.0)	96.5 (92.9 to 98.6)
Positive predictive value, % (95% CI)	63.9 (55.8 to 71.4)	93.0 (86.1 to 97.1)
Negative predictive value, % (95% CI)	98.6 (95.1 to 99.8)	96.0 (92.3 to 98.3)
Positive likelihood ratio (95% CI)	3.48 (2.78 to 4.36)	26.2 (12.62 to 54.31)
Negative likelihood ratio (95% CI)	0.03 (0.01 to 0.11)	0.08 (0.04 to 0.16)

The tradeoffs between true-positive and false-positive rates are shown by presenting the data as a traditional ROC curve (Fig. 2d). The proportional areas under 3 curves (AUC) comparing acute IS vs healthy control and vascular risk factors (all controls), vs non-stroke, and vs all controls and non-stroke yielded in close-up values of 0.914–0.930.

## Discussion

The availability of a sensitive biomarker assay has significant implication for stoke patients. Timely assessment of ischemic stroke requires attention from multidisciplinary medical specialists and nurses (primary care, heart and vascular service, anesthesiology, and emergency medicine) who are the first to meet patients suspected of having a stroke. A rapid and accurate assessment of acute IS in a patient who presents with symptoms suggestive of transient ischemic attack/stroke to a primary care physician or nurse/resident or at the ED is imperative for providing the best possible and most timely care. It is especially needed, when MRI is contraindicated, as is the case in a number of patients due to metal implants, medical instability, obesity, and/or severe claustrophobia. A biomarker for acute IS may help to diagnose and manage these patients more safely and effectively [12].

It has been proposed that biomarkers of neurotoxicity and oxidative stress may be indicative for the assessment of cerebral ischemia [13]. Recently it was shown that *N*-methyl-D-aspartate (NMDA) excitatory receptors which are located on the surface of microvessels, regulate vasoconstriction/vasodilatation, and subtle neurovascular dysfunctions [13], [14]. The NR2 peptide has also been recently identified as a plasma biomarker for acute cerebral ischemia [15]. During the acute phase of the ischemic cascade, a massive release of glutamate up-regulating excitotoxic NMDA receptors has been detected [16], in particular, from endothelial cells of cerebral microvessels [14]. The N-terminal fragments of the NR2-subunit are rapidly cleaved by serin-proteases and released into the bloodstream, where they can be detected directly as NR2 peptide fragments with a molecular weight of about 5 kD [15].

The diagnostic yield of NR2 peptide measurements in blood when used in conjunction with a detailed neurological assessment and radiological evaluation of ischemic stroke patients has been recently reported as a case report study (see File S1) [17]. To explore further the NR2 peptide diagnostic potential in bigger size population, a suitable clinical indication should be considered. The present study was focused on performance characteristics of NR2 peptide biomarker assay to differentiate patients with acute ischemic stroke from all other non-stroke (presented with acute stroke symptoms but had no stroke, stroke mimics, vascular risk factors, and healthy controls).

To clarify the critical cutoff value for NR2 peptide concentrations, four distinct control groups were studied: healthy volunteers, individuals with controlled vascular risk factors, individuals presenting with acute stroke-like symptoms who did not have a stroke and patients with acute stroke (TIA). The analysis of NR2 peptide value variance in patients with acute IS and control group (healthy individuals and persons with controlled vascular risk factors) yielded in statistically significant (P<0.0001) increase of NR2 peptide values in acute IS.

Data analyses yielded two cutoff values, 0.5 ug/L and 1.0 ug/L. Some healthy individuals showed increase in their NR2 peptide of 1.10–1.15 ug/mL. This may be due to unreported chronic inflammatory disease. Autoimmune disorders (lupus erythematosus, rheumatoid arthritis) neurovasculitis, and renal disorders may have elevated NR2 peptide in certain cases and because NMDA receptors located on the surface of microvessels, they should respond to inflammation [18]. It is well known that in some cases these disorders have a high risk for stroke. The case of probable neurovasculities resulted in ischemic stroke has been described recently in case report study (see File S1) [17]. Also individuals with vascular risk factors such as diabetes and hypertension showed elevated NR2 peptide levels (up to 1.54 ug/L) and NR2 antibodies [17], [19] that may indicate microcirculatory dysfunction in this group.

We also demonstrated the statistically significant increase in NR2 peptide in acute IS compared to non-stroke (P<0.0001). In 3 patients who presented with acute stroke symptoms but had no stroke the NR2 peptide concentrations were above the 1.0 ug/L threshold. One of these patients had migraine (6.84 ug/L), one suffered seizures (1.1 ug/L) and had prior TIA and stroke (>1 year). The third one was diagnosed with meningitis (1.3 ug/L). This is a potential limitation of the use of the assay and therefore should be always used in conjunction with clinical assessment and followed up by imaging studies.

While a moderate correlation of NR2 peptide values and new ischemic cortical lesion with stroke volumes below 200 mL was observed, several patients with acute IS (new cortical lesion of 5–50 mL) had NR2 peptide concentrations below optimal cut off of 1.0 ug/L. This could be attributed to a number of reasons: (i) blood sample was drawn from patient beyond 24 h of symptom onset when biomarker values are already diminishing, (ii) NR2 biomarker might be sensitive to oligemia in the ischemic penumbra and probably, do not respond to necrosis in ischemic core [20], (iii) NR2 peptide values may depend on origin of stroke (thrombotic, embolic, occlusion of small blood vessels or microvessels), and (iv) it might be a false negative result. Further investigations of the biomarker in hyper-acute and acute stroke implying advanced DWI/MRI (3T and higher resolution) might clarify the nature of low NR2 peptide values.

Measurement of the NR2 peptide with a threshold of 1.0 ug/L for patients with symptoms suspected of ischemic stroke presenting within 72 h of symptom onset, may have a potential clinical indication: to assist in the emergent diagnosis of ischemic stroke vs non-stroke prior other diagnostic procedures will be assigned. This premise is supported by the predictive value of the test for recognizing individuals with acute cortical and subcortical IS (93% at 1.0 ug/L cutoff; likelihood ratio, 26.2) and preliminary assessed correlation of biomarker with cortical lesions where NMDA receptors are located. Conversely, the test could be used for ruling out individuals without acute IS. If the test at a cutoff point of 1.0 ug/L were negative, the probability in a posttest for IS would be low (about 4%). The latter could be an approach to speed up ruling out non-stroke within first hours after the onset, when NR2 peptide values were the highest.

In this study biomarker was measured in a single blood draw taken on admission from all participants. It was not intended to examine the effect of medication on the NR2 peptide levels. However, interference of some medication (thrombolytics and anticoagulants) with the biomarker values has been registered and should be explored additionally.

Obtained data suggest that the NR2 peptide could serve as a brain specific biomarker for assessment of acute ischemic stroke. Combined with clinical assessment and neuroimaging it has a potential to differentiate IS from non-stroke in ED. Further clinical studies of the NR2 peptide could expose other possible clinical utilities.

### Study limitations

Certain conditions such as brain tumors, autoimmune diseases, or acute CNS infection may result in increased NR2 peptide levels, resulting in false positive diagnoses. Future investigations analyzing the NR2 peptide concentration in these patients and comparing them with brain MRI will help estimate the potential of false positive NR2 assays. Although these conditions are relatively easy to differentiate clinically, this potential limitation will require detailed assessment.

It is possible that the NR2 peptide is not able to detect certain strokes. Specifically, the detection limit for small strokes or small noncortical IS is currently unknown. In our preliminary clinical studies, we could detect small cortical strokes (lesion size: 1–2 mL, n = 14); however, small lacunar subcortical strokes did not always result in elevated NR2 peptide levels and may at times go undetected. The sensitivity of MRI for small infarcts also at times is limited and a future study needs to compare the positive predictive values of the NR2 peptide with DWI/MRI and clinical assessments. Additionally, thus far we have only limited data to know whether the NR2 peptide becomes elevated within the hyperacute phase of IS (1–3 h of symptom onset).

A body of MRI-based analyses of lesion size should be performed simultaneously with biomarker detection to estimate the preliminary correlation between lesion size and NR2 peptide levels demonstrated here. There may be a significant time lag between blood draw and MRI (24–72 h), which could confound findings. A study of a subpopulation of patients who presented early after stroke symptom onset (within 3 h) and who had rapid advanced imaging to gain a first glimpse into the relation between dynamic changes in brain tissue perfusion, penumbra size, and their effect on biomarker levels would be needed. This information may be useful for future studies using the biomarker to assess stroke risk, outcome, and to help with the selection of acute stroke interventions.

Stroke is different from a TIA and if group TIA and stroke together, cutoffs and all results may be affected. Significant number of TIA patients with symptoms still presented (within 1 h of onset) should be enrolled in separate investigation and a statistical analysis should be performed to eliminate all biases.

Multiple group comparisons also raise the awareness with the attention focused on the strongest differences among all comparisons that are made. As more groups are considered, it becomes more likely that the test may appear to be an improvement over existing methods in terms of at least one symptom. As the number of comparisons increases, it becomes more likely that the groups being compared will appear to differ in terms of at least one attribute. However a difference between the groups is only meaningful if it generalizes to an independent sample of data (e.g. to an independent set of people tested with the same assay).

## Supporting Information

File S1 **Case report study paper by Weissman JD, Khunteev GA, Dambinova SA (2012) Biomarkers in acute stroke. MAG J 3: 20–22.**
(PDF)
