# Correction: Diagnostic Potential of the NMDA Receptor Peptide Assay for Acute Ischemic Stroke

**DOI:** 10.1371/annotation/6130c605-086b-46af-8f6f-6c76b8eb9c84

**Published:** 2013-03-05

**Authors:** Svetlana A. Dambinova, Kerstin Bettermann, Theodore Glynn, Matthew Tews, David Olson, Joseph D. Weissman, Richard L. Sowell

In our recent article 'Diagnostic potential of the NMDA receptor peptide assay for acute ischemic stroke', we did not declare several potential competing interests relevant to this work. We apologize for this omission and would like to disclose the following information:

More than 10 years ago Dr. Dambinova filed three patents related to novel NMDA receptor biomarkers and methods for transient ischemic attack/ischemic stroke assessment and transferred these to CIS Biotechc Inc., of which she was a scientific director at the time.

CIS Biotech, Inc. donated the NR2 peptide assay reagents employed in the study. Joseph Weissman is a medical consultant for this company. The study was not funded by CIS Biotech and the company does not currently develop any products related to the results described in the article.

Dr. Dambinova has received travel expenses for participation at lectures from the following companies: Roche Diagnostics, Bayer Diagnostics, Siemens Medical Solution, Abbott, Pfizer . She has performed phone consultations to Phillips, Denka Seiken, and Merck free of charge. 

