# Supplementary material for: Diagnostic Potential of the NMDA Receptor Peptide Assay for Acute Ischemic Stroke
Source: PLoS One. 2012 Jul 27;7(7):e42362. doi: 10.1371/journal.pone.0042362 (PMC3407099; doi:10.1371/journal.pone.0042362)
Supplement: File S1 — Case report study paper by Weissman JD, Khunteev GA, Dambinova SA (2012) Biomarkers in acute stroke. MAG J 3: 20–22. (PDF) [file pone.0042362.s001.pdf]

Biomarkers in acute stroke

By Joseph D. Weissman, M.D., Ph.D, DeKalb Medical Center; German A. Khunteev, M.D., Ph.D., CIS Biotech Inc.; Svetlana A. Dambinova, Ph.D, D.Sc., Kennesaw State University

These cases are presented to illustrate the potential utility of novel biomarkers to enhance the diagnosis and guide treatment of acute stroke. Currently, no biomarkers (including those discussed here) are FDA-approved and all are still in clinical development. The goal is to demonstrate the potential utility of NR2 biomarkers in the diagnosis and treatment of stroke.

The NMDA receptor is the major excitatory amino acid receptor in the central nervous system and is a tetramer of 2 NR1 and 2 NR2 subunits.[24] Ischemia produces an excitotoxic process, which is the pathological process where nerve cells are damaged and undergo apoptosis stimulated by excessive neurotransmitters like the amino acid glutamate. During this process, NR2 receptors are degraded and short peptides are released (NR2 peptides) that can be detected in the bloodstream.[25] These peptides also induce the formation of autoantibodies (NR2Ab) which together have been proposed as biomarkers of the neurotoxicity underlying cerebral ischemia and stroke.[13-15] The following cases are taken from our ongoing research into the application of NR2 biomarkers on acute stroke at DeKalb Medical Center.

Case 1:

A 66-year-old woman presented with a history of hypertension, cardiomegaly and a multiple recent transient episodes of L hemiparesis over the prior three months. She had a similar episode on the day of admission and was brought by her family to the emergency room. As she presented less than three hours post symptom onset, she was screened for intravenous recombinant tissue plasminogen activator (rTPA) use with head CT (negative for acute changes but showing signs of chronic stroke); other screening tests were also negative. She was within minutes of receiving intravenous rTPA when her hemiparesis completely cleared, making her ineligible for rTPA. As part of an ongoing clinical trial of NR2 biomarkers a blood sample taken while she still had the neurological deficit showed elevated NR2 antibodies and peptides[23] (Table 1). She was admitted for observation and had no deficits until another episode of hemiparesis occurred 10 hours later. Due to the time elapsed and other factors, she did not qualify for TPA under the then current criteria. This second deficit did not clear. MRI showed a massive R hemispheric infarction on diffusion weighted and T2-weighted magnetic resonance imaging (Figure 1). There were further elevations in both NR2 peptide and NR2 antibody levels (Table 1) following completed infarction.

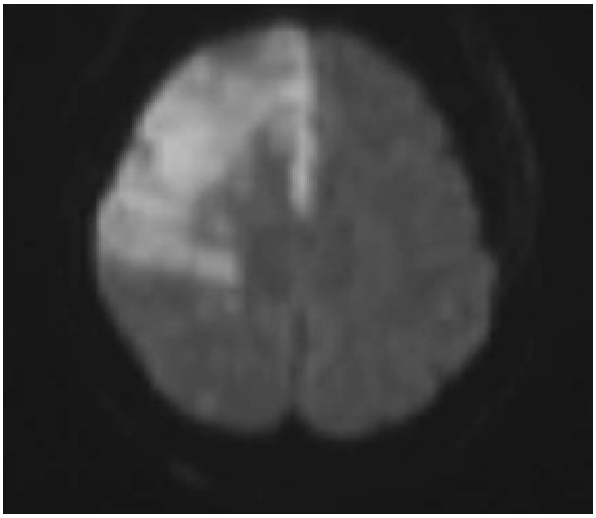

Fig 1 a – Diffusion weighted MRI

Table 1

Time Sample Drawn	NR2 Peptide	NR2 Antibody
**During TIA:**	9.1 ng/ml	13.4 ng/ml
**Post Infarction:**	20.5 ng/ml	14.9 ng/ml
**[upper limit of normal]**	0.5 ng/ml	2.0 ng/ml

This case illustrates the potential utility of the NR2 peptide assay in detecting ischemia. The NR2 peptide was elevated during the initial transient deficit. This supports that the NR2 peptide test may be positive at an early point in the ischemic process where changes are reversible. In this case, the patient recovered initially from her deficit only to have a second event. At the time that this case occurred TPA guidelines did not support the use of rTPA 12 hours after onset of the initial deficit. The treatment of fluctuating strokes with rTPA remains controversial. The observation that the NR2 peptide response to stroke is transient suggests that it could be used as a surrogate for the requirement that rTPA be used not later than 3-4.5 hours after the onset of stroke. This of course would require clinical trials for confirmation. In the case of fluctuating stroke as in the Case 1, the combination of diffusion-weighted MRI, clinical data and biomarker data might help guide TPA use. This would also require confirmation in the form of clinical trials.

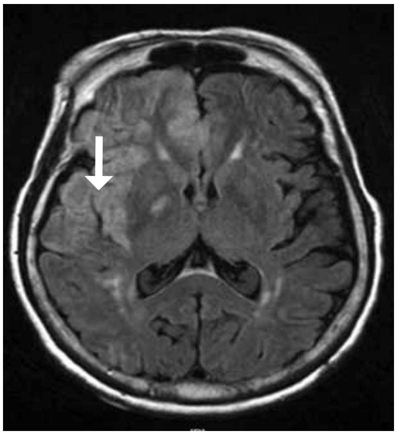

Fig 1 b – FLAIR MRI - Region of stroke

Case 2:

A 21-year-old woman presented with headaches and visual complaints and a T2- weighted MRI scan showed multiple bilateral subcortical areas of white matter abnormality. This was followed by a worsening in condition that resulted in admission to the hospital with left hemiparesis and delirium. At about this time it was revealed by her family that she abused "crystal" methamphetamine. A CT scan after admission showed acute R hemispheric infarction and MRA showed diffuse vasospasm of cerebral vessels without the beading associated with typical vasculitis (Figures 2 and 3). TPA was not applicable because of a delay in presentation to the hospital among other reasons. She received treatment including steroids and made significant recovery, albeit with residual hemiparesis.

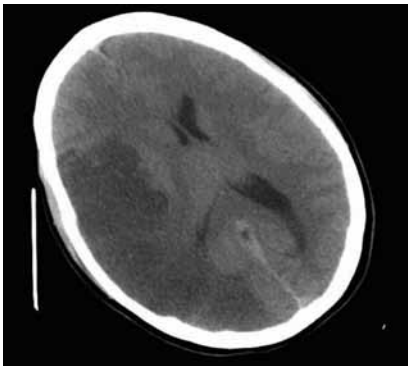

Figure 2 – CT scan

NR2 peptide levels in this case obtained in the course of our research protocol on acute stroke showed very significant and prolonged elevation for over six days, a pattern not seen in ischemic stroke (Figure 4). Based on current experience with the NR2 peptide, these results suggest an ongoing ischemic process. Repetitive or ongoing infarction would not be unexpected with prolonged vasospasm or vasculitis, but would be unusual for a typical stroke. This suggests that processes like vasculitis or vasospasm could be monitored with serial NR2 peptide measurements or that serial measurements might assist in the diagnosis of these challenging disorders.

Discussion

Despite advances in treatment and diagnosis, acute stroke remains difficult to manage clinically. Current diagnosis and treatment relies heavily on patient history for the time of onset which is currently defined as the time that the patient was last known to be intact. Additional diagnostic modalities as well as therapies may be necessary for further improvements in stroke care. There is great interest in blood tests (biomarkers) which may provide additional information regarding stroke risk or the detection of acute stroke. It is understood that any biomarker would have to be validated in controlled clinical trials similar to those used for drug development where initial research is based in basic science and limited patient studies in order to set up large controlled clinical studies.

Troponin is a well-established biomarker for myocardial ischemia, but there is no similarly useful stroke biomarker that has been validated in clinical testing and widely used in current clinical practice. Several biomarkers for detection of stroke risk and/or acute stroke have been investigated. These include anti-phospholipid antibodies, anti-cardiolipin antibodies, von Willebrand factor,[4,5] C-reactive protein (CRP), phospholipase A2 (Pl-PLA2),[6] matrix metalloproteinase-9 (MMP-9),[7] VCAM,[8] homocysteine,[9] glutamate, neuron-specific enolase, myelin basic protein, S-100b,[10,11] B-type neurotrophic growth factor, monocyte chemotactic protein-1[4,5,12] and others. These biomarkers have low predictive value for stroke in the near term and/or reflect late stages in the development of a completed stroke. What is needed is a biomarker that reflects the early stages of ischemia or oligemia that are potentially reversible or more treatable in the r-TPA time frame. Such a biomarker might also be used to potentially determine if a patient with fluctuating stroke is suitable for thrombolysis.

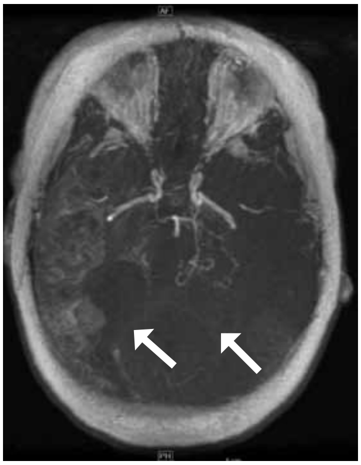

Figure 3 - MRA brain - Vasospasm creates large areas of the brain with no visible vessels

Preliminary studies of the NR2 biomarkers supports their utility in detecting stroke as does their association with the early stages of excitoxicity. Following acute stroke, the NR2 peptide level rapidly increases and then rapidly decreases.[16] The NR2 peptide increases in association with microemboli during carotid endarterectomy and with postoperative neurological deficits following carotid endarterectomy[16, 17] and correlates with infarct volume in acute cortical strokes.[18] It does have limitations in that strokes less than 2-3 ml in volume or involving only white matter areas may not produce detectable NR2 peptide fragments; and that the peptides are very labile and degrade rapidly at room temperature in standard collection vials. In clinical experience the sensitivity of the NR2 peptide for infarcts over 2 ml in volume is 93 percent in strokes with onset with time of onset less than six hours. If small lacunar strokes are included, the sensitivity overall will depend on the number of patients within in the studied population who have the smaller strokes.

IgG autoantibodies to the fragments of the NMDA receptor have been recognized in the serum of stroke patients.[19] In contrast to the very labile NR2 peptides, the NR2 autoantibodies are stable and can be detected using standard blood collection procedures and ELISA detection methods. These antibodies are present in the aftermath of stroke and persisted for a period of many months, and may have other clinical applications.[20] Perioperative serum concentrations of NR2Ab have been shown to be predictive of severe neurological adverse events after cardiac surgery.[21] Patients with a positive NR2Ab level >2.0 ng/mL

(continued on page 22)

(continued from page 27)

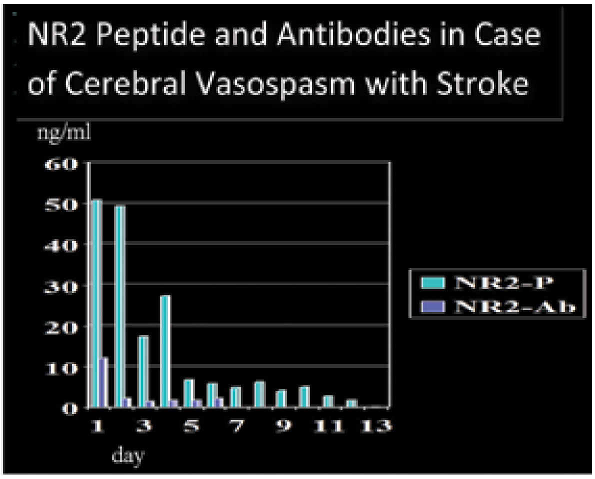

Figure 4. NR2 peptide and antibody levels

prior to surgery were nearly 18 times more likely to experience a postoperative neurological event than patients with a negative test.[21] In patients with a history of a preceding stroke, or TIA events, NR2 antibodies were elevated compared to patients with first-time events and controls.[22] Some elevated NR2 antibody levels are detected in patients without a history of stroke but with a higher risk of subclinical stroke or "age-related" white matter changes. Whether this reflects subclinical stroke activity or is simply a false positive is a subject under current investigation.[22]

Conculsion

These cases are anecdotal and involved relatively unusual clinical situations of stuttering stroke onset and amphetamine-associated cerebral vasospasm. I have chosen them because they demonstrate how biomarkers might be able to assist diagnosis and treatment in stroke. The point is that they illustrate the potential impact of biomarker-based information on clinical stroke management. At this point in time cerebral vasospasm and stuttering stroke present challenges in both diagnosis and management. If these findings are supported with future studies, NR2 peptide and antibody levels may ultimately provide information by which therapy can more directly address these difficult conditions.

References

1. Vermeer SE, Den Heijer T, Koudstaal PJ, Oudkerk M, Hofman A, Breteler MM Incidence and risk factors of silent brain infarcts in the population-based Rotterdam Scan Study. Stroke 2003 34(2):392-6.

2. Saposnik G, Noel de Tilly L, Caplan LR. Pontine warning syndrome. Arch Neurol. 2008 Oct;65(10):1375-7.

3. Lee J, Albers GW, Marks MP, Lansberg MG. Capsular warning syndrome caused by middle cerebral artery stenosis. J Neurol Sci. 2010 Sep 15;296(1-2):115-20. Epub 2010 Jul 8.

4. Brey RL, Abbott RD, Curb JD, Sharp DS, Ross GW, Stallworth CL, et al. beta(2)-Glycoprotein 1-dependent anticardiolipin antibodies and risk of ischemic stroke and myocardial infarction: the Honolulu Heart Program. Stroke 2001;32(8):1701-6.

5. Tuhrim S, Rand JH, Wu X, Horowitz DR, Weinberger J, Goldman ME, et al. Antiphosphatidyl serine antibodies are independently associated with ischemic stroke. Neurology 1999;53(7):1523-7.

6. Ballantyne CM, Hoogeveen RC, Bang H, Coresh J, Folsom AR, Chambless LE, et al. Lipoprotein-associated phospholipase A2, high-sensitivity C-reactive protein, and risk for incident ischemic stroke in middle-aged men and women in the Atherosclerosis Risk in Communities (ARIC) study. Arch Intern Med 2005;165(21):2479-84.

7. Peters DG, Kassam A, St Jean PL, Yonas H, Ferrell RE. Functional polymorphism in the matrix metalloproteinase-9 promoter as a potential risk factor for intracranial aneurysm. Stroke 1999;30(12):2612-6.

8. Blum A, Khazim K, Merei M, Peleg A, Blum N, Vaispapir V. The stroke trial - can we predict clinical outcome of patients with ischemic stroke by measuring soluble cell adhesion molecules (CAM) Eur Cytokine Netw 2006;17(4):295-8.

9. Haapaniemi E, Helenius J, Soinne L, Syrjala M, Kaste M, Tatlisumak T. Serial measurements of plasma homocysteine levels in early and late phases of ischemic stroke. Eur J Neurol 2007;14(1):12-7.

10. Jauch EC, Lindsell C, Broderick J, Fagan SC, Tilley BC, Levine SR. Association of serial biochemical markers with acute ischemic stroke: the National Institute of Neurological Disorders and Stroke recombinant tissue plasminogen activator Stroke Study. Stroke. 2006;37(10):2508-13.

11. Hill MD, Jackowski G, Bayer N, Lawrence M, Jaeschke R. Biochemical markers in acute ischemic stroke. CMAJ 2000;162(8):1139-40.

12. Reynolds MA, Kirchick HJ, Dahlen JR, Andberg JM, McPherson PH, Nakamura KK, et al. Early biomarkers of stroke. Clin Chem 2003;49(10):1733-39.

13. Dambinova SA, Khounteev GA, Skorometz AA. Multiple panel of biomarkers for TIA/stroke evaluation. Stroke 2002; 33(5):1181-2.

14. Dambinova SA, Khounteev GA, Izykenova GA, Zavolokov IG, Ilyukhina AY, Skoromets AA. Blood test detecting autoantibodies to N-methyl-D-aspartate neuroreceptors for evaluation of patients with transient ischemic attack and stroke. Clin Chem 2003;49(10):1752-62.

15. Gascón S, Sobrado M, Roda JM, Rodríguez-Peña A, Díaz-Guerra M. Excitotoxicity and focal cerebral ischemia induce truncation of the NR2A and NR2B subunits of the NMDA receptor and cleavage of the scaffolding protein PSD-95. Mol Psychiatry 2008;13(1):99-114.

16. Dambinova SA. Biomarkers for transient ischaemic attack (TIA) and ischaemic stroke. Clin Lab Int 2008;32:7-11.

17. Brightwell RE, Khunteev GA, Cheshire NJ, Dambinova SA. NR2 Peptide Correlates with Acute Ischemic Events after Carotid Revascularization (manuscript in preparation).

18. Weissman et al, 2007) JD Weissman, G Khunteev, R Heath, G Izykenova, S Dambinova. NR2 peptide levels correlate with acute cortical stroke volume and may reflect reversible ischemia. P31.157. American Stroke Association International Stroke Conference, San Francisco, California 8, Feb. 2007.

19. Dambinova SA. Glutamate Neuroreceptors. Leningrad: Nauka, 1988 in Russian.

20. Gusev EI, Skvortsova VI, Izykenova GA, Alekseev AA, Dambinova SA. The level of autoantibodies to glutamate receptors in the blood serum of patients in the acute period of ischemic stroke. In Russian. Zh Nevropatol Psikhiatr Im S S Korsakova. 1996;96(5):68-72.

21. Bokesch PM, Izykenova GA, Justice JB, Easley KA, Dambinova SA. NMDA receptor antibodies predict adverse neurological outcome after cardiac surgery in high-risk patients. Stroke. 2006;37:1432-36.

22. Weissman JD, Khunteev GA, Heath R, Dambinova SA. NR2 antibodies: risk assessment of transient ischemic attack (TIA)/stroke in patients with history of isolated and multiple cerebrovascular events. J Neurol Sci. 2011 300:97-102.

23. Gold Dot Peptide and Antibody ELISA Tests, CIS Biotech, Atlanta, GA.

24. Dingledine R, Borges K, Bowie D, Traynelis SF . The glutamate receptor ion channels. Pharmacol. Rev. 1999. 51: 7–61

25. Dambinova SA, Izykenova GA, Khounteev GA, Skoromets AA. Brain NR2 peptide in blood assay for stroke assessment ruling out hemorrhage. J Stroke Cerebrovasc Dis. 2003 12: 248.
